# Transosseous Equivalent Meniscus Allograft Transplantation Using an All‐Suture‐Based Anchor Technique

**DOI:** 10.1002/atn2.70278

**Published:** 2026-10-22

**Authors:** Megan E. Barney, Mikalyn T. DeFoor, Lily Swope, Trevor McBride, Emily R. McDermott, David J. Tennent

**Affiliations:** ^1^ San Antonio Military Medical Center San Antonio Texas U.S.A.; ^2^ Evans Army Community Hospital Fort Carson Colorado U.S.A.

## Abstract

Meniscal allograft transplantation is a surgical solution for symptomatic meniscal deficiency aimed at restoring native knee biomechanics and tibiofemoral contact pressures with the goal of slowing the progression of osteoarthritis. Anatomic fixation of the meniscal root is essential to a successful outcome as this normalizes native tibiofemoral contact pressures, restores hoop stresses, and improves graft survivorship. This is a technically challenging procedure with various techniques described that focus on the use of osseous and soft tissue‐only fixation strategies. This technical note describes an all‐inside method of posterior and anterior meniscal root fixation in the setting of a medial meniscal allograft transplantation using an all‐suture‐based transosseous equivalent fixation strategy that provides anatomic fixation while minimizing the tunnel and implant convergence concerns that often occur during concomitant realignment or instability surgery.

## Graphical abstract

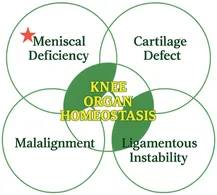

**VIDEO 1:** This technical note shows a transosseous‐equivalent all‐soft‐tissue meniscal allograft transplantation using an all‐suture‐based anchor technique. This patient is a 33‐year‐old active‐duty woman with chronic medial knee pain in the setting of a prior irreparable root‐adjacent degenerative medial meniscus tear. She had exhausted conservative therapy without improvement in her symptoms. Therefore, after shared decision‐making, the plan was to proceed with left knee arthroscopy and medial meniscus allograft transplantation. Preoperative radiographs showed no advanced arthritic changes and neutral coronal alignment bilaterally. Advanced magnetic resonance imaging showed a complex, degenerative tear approximately 1 cm from the posterior root attachment of the medial meniscus, otherwise with a well‐preserved articular surface of the medial compartment. The procedure begins with diagnostic arthroscopy to confirm the functionally deficient medial meniscus and preserved articular surface of the medial compartment. An extracapsular high, proximal posteromedial portal was established under direct visualization using an outside‐in technique. A more proximal‐than‐standard portal is essential to ensure safe and appropriate positioning of the subsequently placed anchor with clearance around the posterior femoral condyle. Once the appropriate portal placement is established, and the all‐suture knotted anchor is placed in the anatomic attachment site of the posterior root of the medial meniscus, a more proximal‐than‐standard posteromedial portal is essential to ensure safe and appropriate positioning of the subsequently placed posterior root anchor. A 7‐mm twist‐in cannula can be utilized to assist with arthroscopic instrumentation and anchor placement. Then, the looped passing sutures are taken through a dilated anteromedial portal staying medial to the fibers of the posterior cruciate ligament. A Micro SutureLasso (Arthrex, Naples, FL) is used to secure the preplaced horizontal mattress sutures from the posterior body of the meniscal allograft to the capsule in an outside‐in fashion utilizing a standard open posteromedial exposure. The posterior root sutures are then passed through the all‐suture anchor with tension sutures shuttled through the posteromedial accessory portal. Simultaneously, a fresh size‐matched medial meniscus allograft is prepared on the back table for implantation, removing the meniscus from its osseous attachments. Sequential tensioning is applied to the allograft traction suture and root sutures to advance the allograft into the knee joint. Next, the sequential vertical mattress sutures are passed, alternating through the superior and inferior leaflets of the posterior and middle thirds of the meniscus in an inside‐out fashion. An additional all‐inside anchor can be used posteriorly to secure the far posterior portion of the meniscus if needed. After the posterior and middle thirds of the meniscal allograft are secured, the knee is placed in maximum flexion, and a second all‐suture anchor is placed at the anteromedial meniscus root attachment through the standard anteromedial portal. The prepassed sutures in the anterior horn of the meniscal allograft are then passed through the soft anchor, tensioned, and tied overtop to complete the anterior root fixation with the knee in 90° of flexion. Finally, to complete fixation, the anterior third of the meniscus is then secured using an outside‐in technique. The posterior root anchor sutures are fixed in the end to finalize appropriate tension. Postoperatively, the patient follows a standard meniscal allograft transplantation physical therapy protocol including 6 weeks of nonweight‐bearing with active range of motion limited from 0° to 90° of knee flexion. Video content can be viewed at https://doi.org/10.1002/atn2.70278.

Meniscal allograft transplantation (MAT) is a suitable surgical option for select patients <50 years of age with symptomatic meniscal deficiency. Mean survival rates are estimated to be 60% at 15 years with mean time to failure of 8.2 and 7.6 years for a medial and lateral MAT, respectively.^1^ Anatomic meniscal root repair is critical to disperse hoop stresses, ensure proper load transmission, preserve native tibiofemoral joint biomechanics, and slow the progression of rapidly developing knee osteoarthritis.^2^

Traditional root fixation methods include transosseous transtibial tunnels with all‐soft tissue, bone plugs, or bone bridge techniques—all with equivalent outcomes.^1^ Although the inside‐out transosseous fixation method provides strong fixation, it can be less desirable and more technically challenging in the setting of a concomitant procedure such as high tibial osteotomy or ligament reconstruction and/or previously placed tunnel hardware because of tunnel positioning considerations. All‐suture anchors offer an alternative method of root fixation that preserves bone stock with a small anchor footprint while eliminating the risk of tunnel or implant convergence during concomitant or staged procedures. Furthermore, the all‐inside meniscal root fixation with all‐suture anchors may simplify suture management, reduce iatrogenic cartilage injury, and facilitate efficient, reproducible meniscal root fixation. The aim of this Technical Note is to describe an efficient, reproducible technique for anatomic anterior and posterior meniscal root fixation illustrated in a medial MAT with an accessory posteromedial portal (Video 1).

## SURGICAL TECHNIQUE

### Preoperative Evaluation and Planning

Indications and contraindications for the surgical treatment of symptomatic meniscal deficiency are illustrated in Table 1.^1^ Meniscal deficiency can often coexist with other knee pathologies such as focal cartilage defects, coronal and/or sagittal malalignment, and ligamentous instability (Figure 1). A high index of the suspicion of concomitant pathology and a comprehensive history and physical examination are required to assess and treat the patient with meniscal deficiency.

**TABLE 1:** Indications and Contraindications for Meniscal Allograft Transplantation

**Indications**	**Contraindications**
Near‐complete/full‐thickness meniscal deficiency	Inflammatory arthritis
• 1‐2.5 cm absence of total meniscal length	
• Irreparable posterior root or root equivalent tears	
<50 yr of age	Skeletal immaturity
High demand, active individual	Active nicotine use
Symptomatic—inability to perform desired level of physical activity	Body mass index >30
Minimal cartilage degradation of associated compartment (Kellgren‐Lawrence grade II or less)	Advanced and diffuse cartilage degradation of the associated compartment (Kellgren‐Lawrence grade III or IV)
Ability to comply with rehabilitation guidelines	Untreated ipsilateral limb malalignment, ligamentous instability or focal cartilage defect

Yr, years.

**FIGURE 1:**
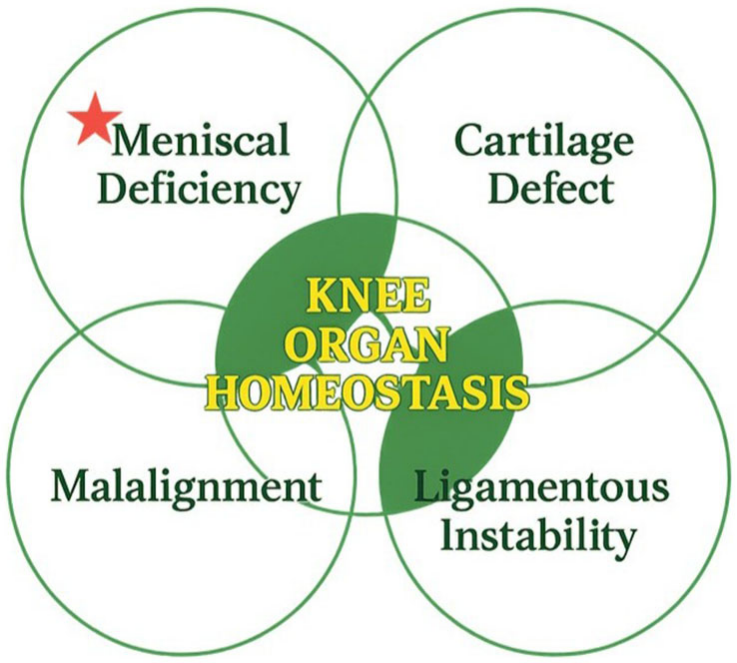
Venn diagram representing the complex interplay of components contributing to the overall homeostasis of a healthy knee organ including the meniscus, cartilage, stability, and coronal/sagittal plane alignment. Star symbol highlights mensical deficiency because that is the focus of this clinical case.

Preoperative evaluation includes plain radiographs of the knee with full‐length standing alignment films (Figure 2). Advanced imaging with magnetic resonance imaging (MRI) is required to more closely assess the integrity of concomitant knee structures and to appropriately diagnose meniscal deficiency (Figure 3). In evaluation of this complex pathology, a diagnostic or “staging” arthroscopy is typically warranted in corroborating the diagnosis of meniscal deficiency and evaluating for additional intra‐articular pathology, as well as confirming the overall health of the articular surface of the joint. Although magnetic resonance imaging is a valuable diagnostic tool, arthroscopy provides direct visualization and is the gold standard for diagnosing intra‐articular pathology and the absence or presence of MAT contraindications.^3^ Once the decision is made to pursue MAT, fresh or fresh‐frozen donor allografts are size‐matched based on either plain films or magnetic resonance imaging measurements.

**FIGURE 2:**
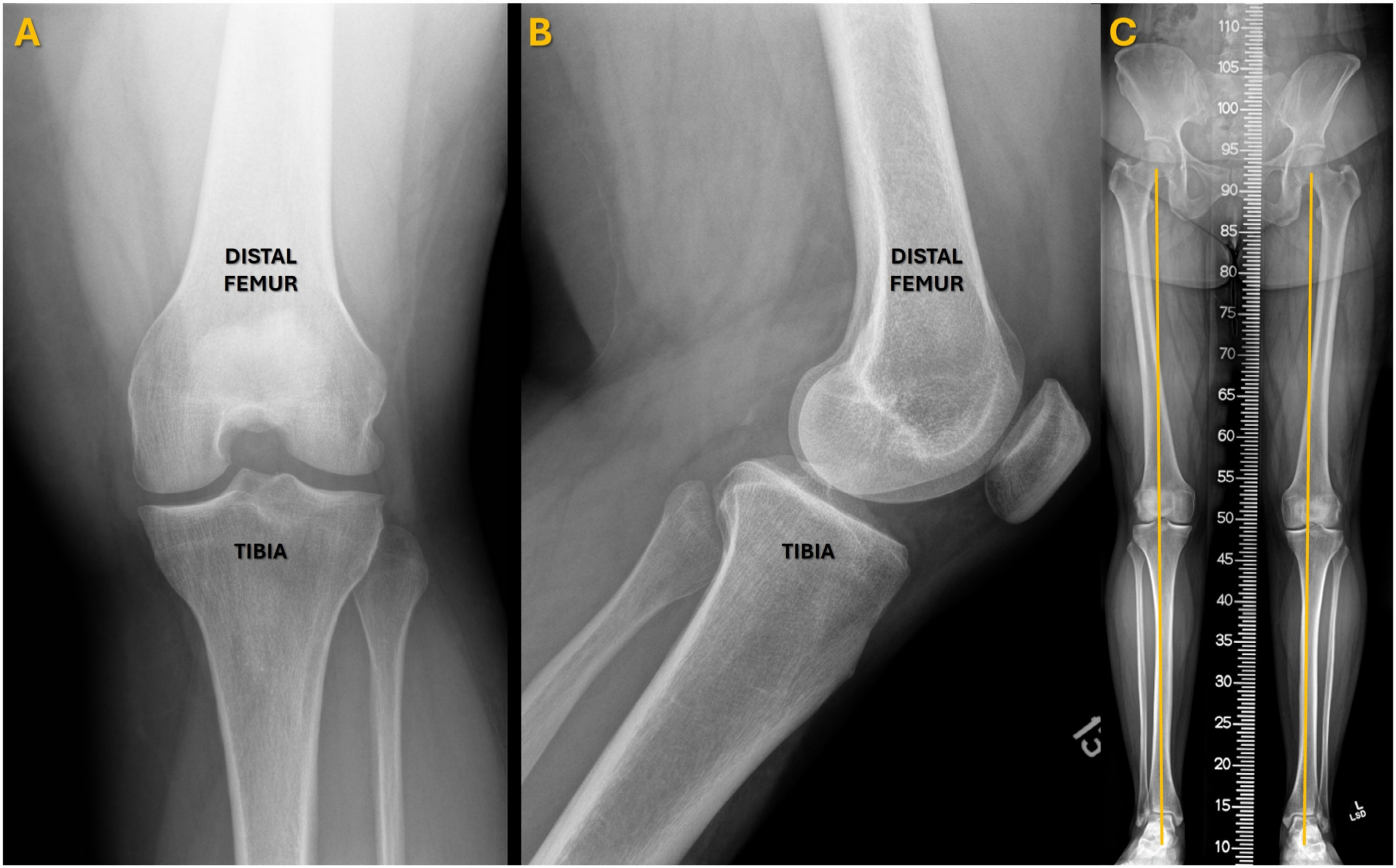
Preoperative weight‐bearing plain radiographs of a left knee including (A) AP, (B) lateral, and (C) full‐length standing alignment films showing intact medial, lateral, and patellofemoral compartments without any significant joint space narrowing and appropriate coronal alignment with slight asymmetric varus on the left. (AP, anteroposterior.)

**FIGURE 3:**
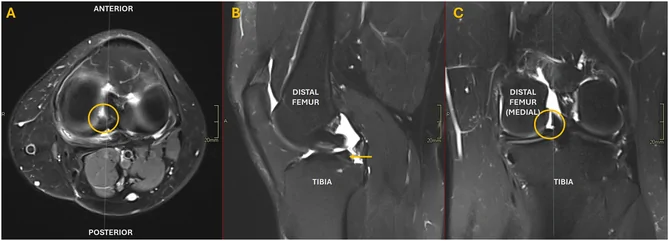
Preoperative T2‐weighted MRI of a left knee including select (A) axial, (B) sagittal, and (C) coronal cuts showing a posterior root‐adjacent medial meniscus deficiency after prior partial medial meniscectomy with diminutive appearance. (MRI, magnetic resonance imaging.)

### Meniscus Evaluation and Debridement

After completion of diagnostic arthroscopy, attention is drawn to the medial compartment to confirm the functionally deficient medial meniscus and ensure lack of advanced osteoarthritis of the medial compartment (Kellgren‐Lawrence grade ≤ II) (Figure 4). Care must be taken to thoroughly debride the remaining meniscal tissue and prepare the meniscal bed for transplantation. To optimize visualization and instrumentation of the medial compartment, trephination of the medial collateral ligament can be performed using an 18‐gauge spinal needle under direct visualization as needed. Percutaneous medial collateral ligament release has been demonstrated to increase the medial compartment width from 5.95 ± 1.32 to 11.09 ± 1.74 mm, almost doubling the working space within the medial tibiofemoral joint, without altering the postoperative rehabilitation protocol.^4^ Using a combination of arthroscopic biters and shaver, the remaining meniscal tissue and roots are trimmed, leaving 1 to 2 mm of the bleeding meniscal rim. It is critical to leave a meniscal rim in place to act as a stable bed for fixation and prevent radial displacement of the allograft.^5^ A meniscal rasp is gently used to abrade the native meniscal bed until punctate bleeding is visualized to stimulate healing.

**FIGURE 4:**
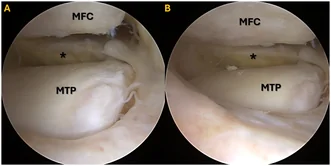
Viewing a left knee positioned in slight flexion with a valgus stress through a standard anterolateral portal with a 30° arthroscope, evaluating with an arthroscopic probe (A) the complete prior subtotal meniscectomy of the medial meniscus (*) and (B) the resulting diminutive appearance of the midbody to the posterior horn of the medial meniscus (*) from the prior partial medial meniscectomy after a complex, irreparable root‐adjacent tear. (MFC, medial femoral condyle; MTP, medial tibial plateau.)

### Graft Preparation

Simultaneously, a fresh size‐matched medial meniscus allograft (AlloSource, Denver, CO) is prepared on the back table for implantation (Figure 5). The meniscus is removed from its osseous attachments. A No. 2 FiberWire (Arthrex, Naples, FL) is used in a Krakow fashion to secure each root with care to terminate each suture end on the inferior portion of the root attachment. An additional No. FiberWire (Arthrex) is placed in a horizontal mattress configuration at the superior portion of the junction of the posterior and middle third of the meniscus. Platelet‐rich plasma is then injected along the periphery of the allograft and then placed into a vancomycin‐soaked sponge.

**FIGURE 5:**
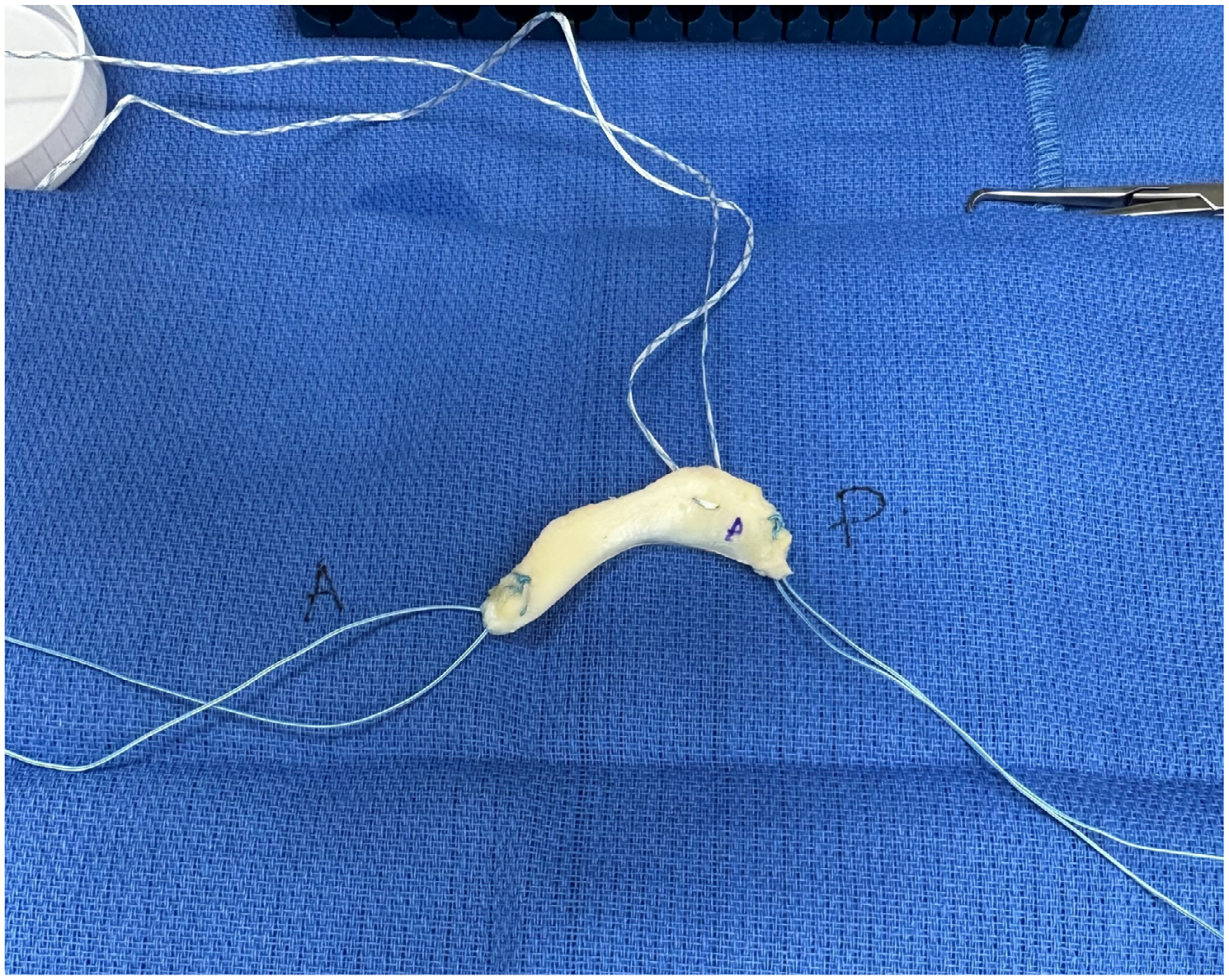
Clinical image of the size‐matched soft‐tissue‐only medial meniscus fresh allograft after preparation with a high‐strength suture whipstitched at both the posterior and anterior horns, in addition to a high‐strength passing suture placed at the junction of the posterior and middle one‐third of the meniscus in a horizontal mattress configuration. (A, anterior; P, posterior.)

### Posterior Root Anchor Fixation

An extracapsular high‐proximal posteromedial portal is established under direct visualization using an outside‐in technique, taking care to avoid neurovascular structures while allowing sufficient excursion around the posterior femoral condyle (Figure 6). A more proximal‐than‐standard portal is essential to ensure safe and appropriate positioning of the subsequently placed anchor. Furthermore, if this portal is placed too anterior, anchor placement is precluded by the posterior femoral condyle, which will limit appropriate placement of the anchor at the anatomic root attachment while also forcing subsequent drilling in a posterior trajectory. When performing concomitant posterior cruciate ligament (PCL) reconstruction, it is preferred that this portal be established with consideration of both procedures and posterior root anchor placement performed prior to PCL graft passage, as this simplifies anchor placement due to posterior tibial translation. Once the appropriate trajectory is confirmed using an 18‐gauge spinal needle, a 7‐mm twist‐in cannula (Arthrex) is placed over a switching stick.

**FIGURE 6:**
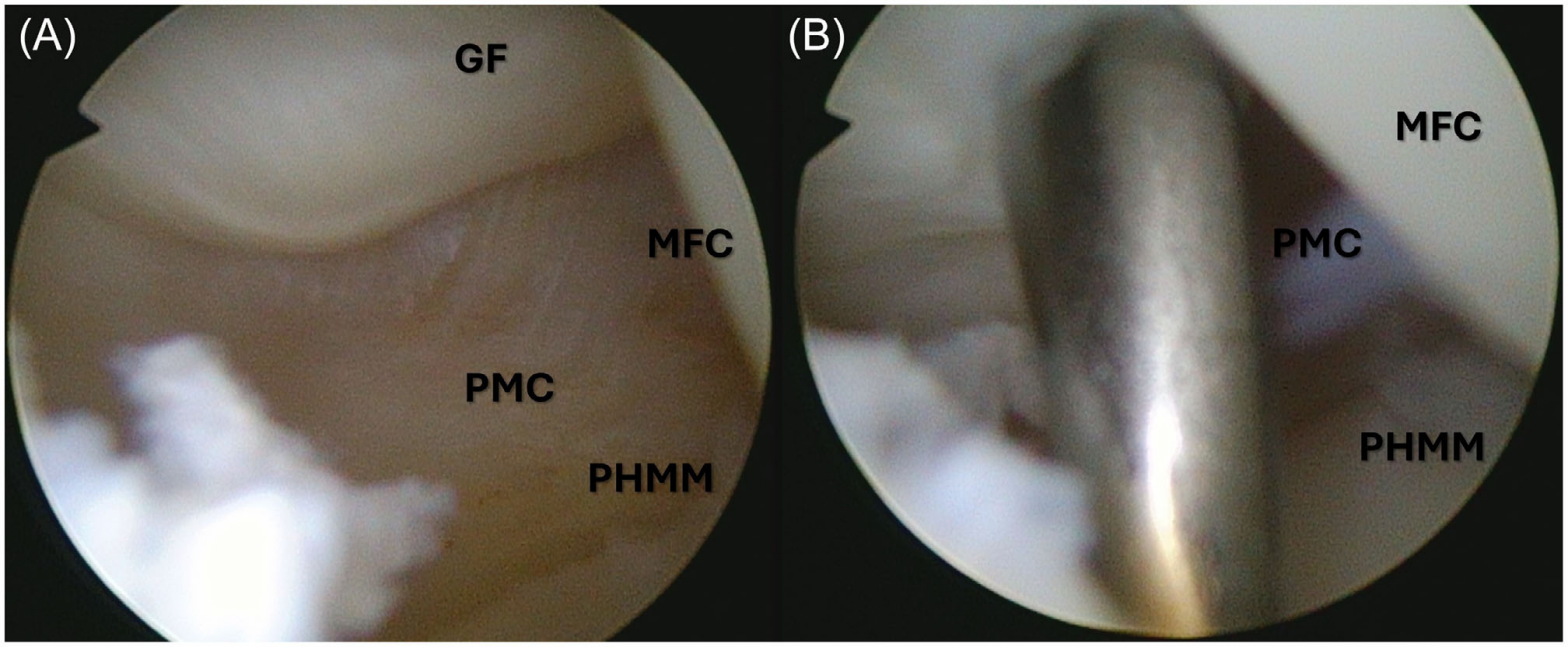
Viewing a left knee positioned in 90° of flexion through a standard anterolateral portal with a 30° arthroscope, a modified Gillquist maneuver is performed to (A) visualize landmarks for the establishment of the posteromedial accessory portal, which is established (B) under direct visualization with an 18‐guage spinal needle followed by a switching stick and a 7‐mm twist‐in cannula. (GF, gastrocnemius fold; MFC, medial femoral condyle; PHMM, posterior horn medial meniscus; PMC, posteromedial capsule.)

Once portal placement is established, an all‐suture‐knotted 2.6‐mm FiberTak Button (Arthrex) is placed at the anatomic attachment site of the posterior root of the medial meniscus, which is 10 mm posterior and 0.7 mm lateral to the medial tibial eminence or the inflection point of the medial tibial plateau articular cartilage with the most proximal portion of the PCL tibial attachment (Figure 7).^6^ Anatomic landmarks are generally visible and used as a guide during arthroscopic visualization. If body habitus prevents the use of a 2.6‐mm FiberTak Button (Arthrex) and the proximal tibia density allows, a 1.8‐mm FiberTak Button (Arthrex) can alternatively be used, as this provides additional implant length for insertion while preserving additional bone stock. The looped, passing sutures are then taken through a dilated anteromedial portal staying medial to the PCL fibers.

**FIGURE 7:**
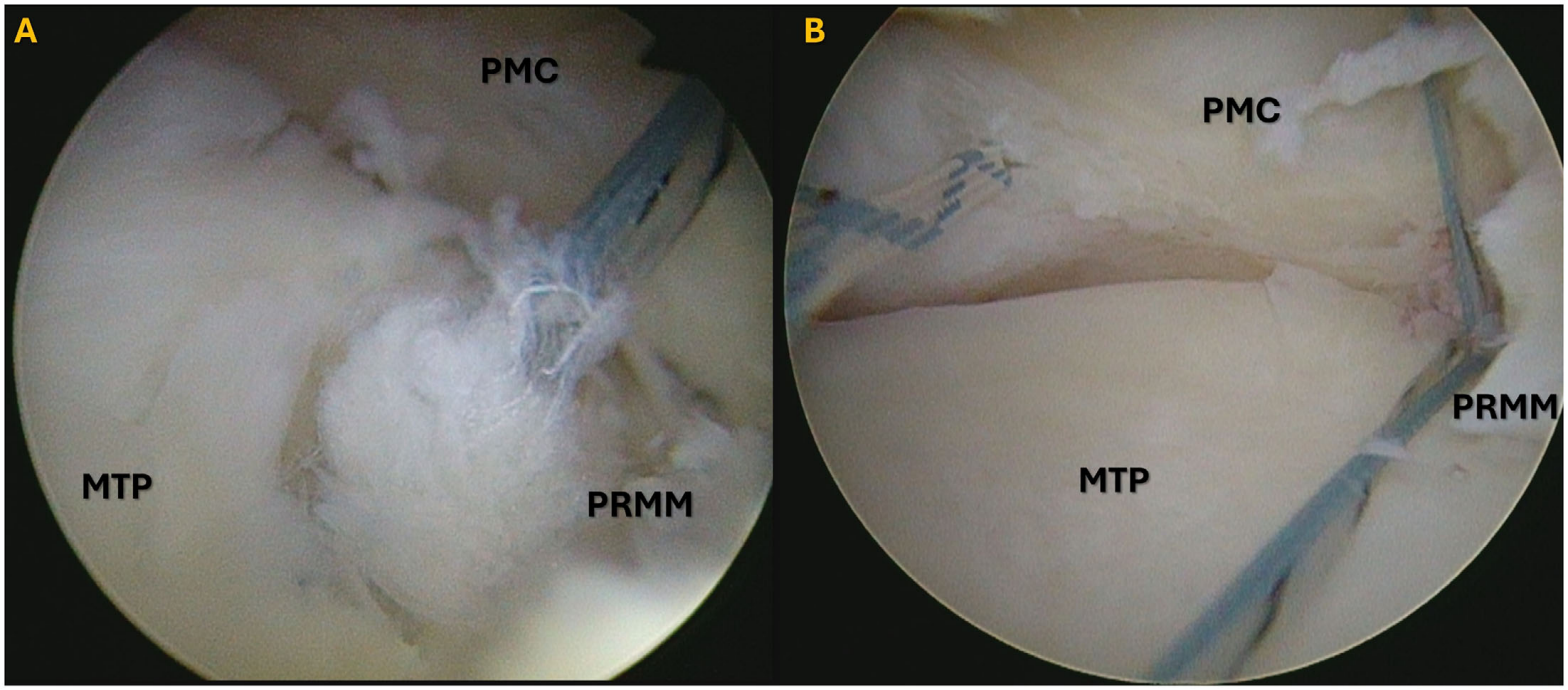
Viewing a left knee positioned in slight flexion with a valgus stress through a standard anterolateral portal with a 30° arthroscope, (A) the all‐suture anchor is placed at the anatomic footprint of the posteromedial meniscal root attachment, (B) followed by an outside‐in to inside‐out fixation of the posterior body with a horizontal mattress configuration to assist with the reduction of the posterior body and the horn of the meniscus prior to securing the root fixation. (MTP, medial tibial plateau; PMC, posteromedial capsule; PRMM, posterior root medial meniscus.)

### Graft Passage and Final Fixation

A Micro SutureLasso (Arthrex) is used to secure the preplaced horizontal mattress sutures from the posterior body of the meniscal allograft to the capsule in an outside‐in fashion utilizing the standard open posteromedial exposure. The posterior root sutures are then passed through the FiberTak Button (Arthrex) with tension sutures shuttled through the posteromedial portal. The allograft traction suture and root sutures are then sequentially tensioned to advance the graft into the knee. Next, sequential vertical mattress sutures are passed alternating the superior and inferior aspects of the posterior and middle thirds of the meniscus in an inside‐out fashion. An additional all‐inside anchor can also be used posteriorly to secure the far posterior portion of the meniscus if needed.

With the knee in maximum flexion, a second 2.6‐mm FiberTak Button (Arthrex) is placed at the anterior medial meniscus root attachment through the anteromedial portal, which has a broad insertion at the anterior intercondylar crest of the tibia just anterior to the anterior cruciate ligament fibers (Figure 8).^7^ The prepassed sutures in the anterior horn of the allograft are then passed through the soft anchor, tensioned, and then tied overtop to complete the anterior root fixation with the knee in 90° of flexion. The posterior root anchor sutures are tied last to ensure appropriate tension and fixation of the posterior root. To complete fixation, the anterior third of the meniscus is then secured using an outside‐in technique. This is performed in the end as visualization is now maximized, and tension is finalized (Figure 9).

**FIGURE 8:**
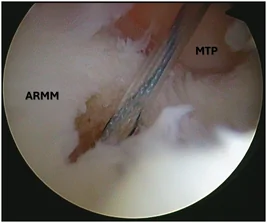
Viewing a left knee positioned in 90° of flexion through a standard anterolateral portal with a 30° arthroscope, the all‐suture anchor is placed at the anatomic footprint of the anteromedial meniscal root attachment, just off the anterior intercondylar crest of the tibia. (ARMM, anterior root medial meniscus; MTP, medial tibial plateau.)

**FIGURE 9:**
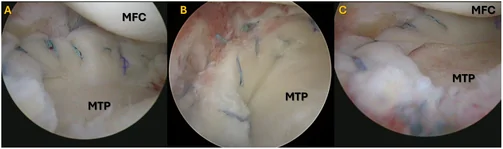
Viewing a left knee positioned in slight flexion with a valgus stress through a standard anterolateral portal with a 30° arthroscope, illustrating the final medial meniscal allograft transplantation fixation including the (A) anterior root, (B) mid‐body, and (C) posterior root with excellent reduction to the capsule and good stability without gapping after final repair. (MFC, medial femoral condyle; MTP, medial tibial plateau.)

## DISCUSSION

Although a technically challenging operation, the ability of a MAT to restore tibiofemoral contact pressures compared with that of native conditions may delay rapidly progressing osteoarthritic changes and ultimately the need for a more invasive joint‐replacing procedure in the symptomatic, meniscal‐deficient knee.^8^ A key technical consideration in MAT is secure and anatomic fixation of the meniscal roots to restore native biomechanics and hoop stresses. As multiple techniques have been described including bone plugs, keyhole techniques, and suture‐based repairs, no single technique has shown clinical superiority.^9^, ^10^, ^11^

Under physiological loading, biomechanical studies in porcine models have shown lower maximum load at failure and lower stiffness, with no difference in displacement during cyclic loading, after posterior medial meniscus root suture anchor fixation, suggesting a mandatory protected weight‐bearing status during the postoperative period.^12^, ^13^ While showing a higher overall load to failure, traditional transosseous tunnel fixation may not always be feasible particularly in the setting of a concomitant osteotomy, ligament reconstruction, and/or a revision surgery—all of which may serve as an obstacle to fixation due to tunnel convergence, suture management, and prior hardware placement. This knotted all‐suture anchor root repair offers an efficient, reproducible all‐inside technique with an implant that is easy to use and familiar to most arthroscopic surgeons that eliminates the risk of tunnel convergence. There are several key advantages and disadvantages, as well as technical pearls and pitfalls, of this surgical technique, which are highlighted in Tables 2 and 3, respectively.

**TABLE 2:** Key Advantages and Disadvantages of Meniscal Allograft Transplantation With All‐Suture Soft Anchor Root Fixation

**Advantages**	**Disadvantages**
Arthroscopic surgeons are familiar with the use of this implant and fixation method for this all‐inside root repair	Surgeons must be familiar with the anatomy and surrounding neurovascular structures of the accessory posteromedial portal
Eliminates potential of tunnel convergence commonly encountered with transosseous root repairs in concomitant ACLR	Additional cost of the all‐suture implant
Easily maneuvered implant around the setting of a concomitant osteotomy site and/or prior hardware placement (i.e., prior failed ACLR)	Technically challenging visualization and retrieval of posterior passing sutures
In large thigh, a 1.8‐mm FiberTak Button (Arthrex, Naples, FL) can be used as an alternate of the 2.6‐mm implant	Fixation likely does not support accelerated weight‐bearing postop protocols due to lower biomechanical maximum load and stiffness in animal models^13^
• Provides additional implant length for insertion	
• Preserves additional bone stock	

ACLR, anterior cruciate ligament reconstruction; postop, Postoperative.

**TABLE 3:** Key Technical Pearls and Pitfalls of Meniscal Allograft Transplantation With the All‐Suture Soft Anchor Root Fixation

**Pearls**	**Pitfalls**
Establish a high, extracapsular posteromedial portal under direct visualization to establish the correct trajectory for posterior root all‐suture anchor placement	An accessory portal that is too anterior/too distal may be blocked by the posterior femoral condyle, forcing a posterior drilling trajectory and risking nonanatomic anchor placement for root fixation
Use the anatomic posteromedial root footprint as the target for anchor placement, just posterior/lateral to the medial tibial eminence, near the PCL tibial attachment	Nonanatomic posterior root fixation may compromise hoop‐stress restoration and graft biomechanics
Sequentially tension the graft traction suture and posterior root sutures to advance the allograft before final fixation	Overtensioning or premature tying may malreduce the graft, create extrusion, or limit final anatomic positioning
Fix the anterior root with the knee in maximal/90° flexion, and then tie the posterior root in the end to finalize overall graft tension	Tying the posterior root too early may prevent appropriate anterior root positioning and final circumferential tensioning

PCL, posterior cruciate ligament.

## DISCLOSURES

The authors (M.T.D., D.J.T.) declare the following financial interests/personal relationships that may be considered as potential competing interests: M.T.D. reports *Arthroscopy Journal* (editorial board member), American Academy of Orthopaedic Surgeons (board or committee member), and Society of Military Orthopaedic Surgeons (board or committee member), outside of the submitted work. D.J.T. reports Arthroscopy Association of North America (board or committee member), and Society of Military Orthopaedic Surgeons (board or committee member), outside of the submitted work. The other authors (M.E.B., L.S., T.M., E.R.M.) declare that they have no known competing financial interests or personal relationships that could have appeared to influence the work reported in this paper.
